# Supplementary material for: A macrophage-predominant immunosuppressive microenvironment and therapeutic vulnerabilities in advanced salivary gland cancer
Source: Nat Commun. 2025 Jun 12;16:5303. doi: 10.1038/s41467-025-60421-0 (PMC12162891; doi:10.1038/s41467-025-60421-0)
Supplement: Supplementary file 1 — Supplementary Information [file 41467_2025_60421_MOESM1_ESM.pdf]

Supplementary Figures

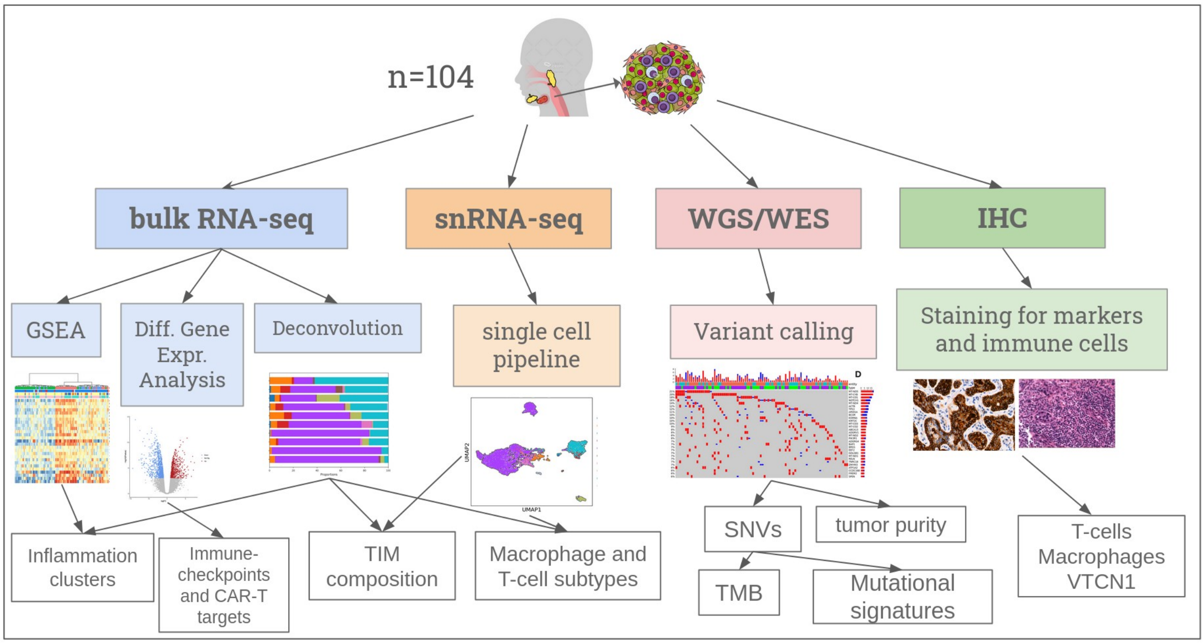

Suppl. Figure 1: *Graphical abstract of datasets and analyses.* Figure created using the Mind the Graph platform (www.mindthegraph.com)

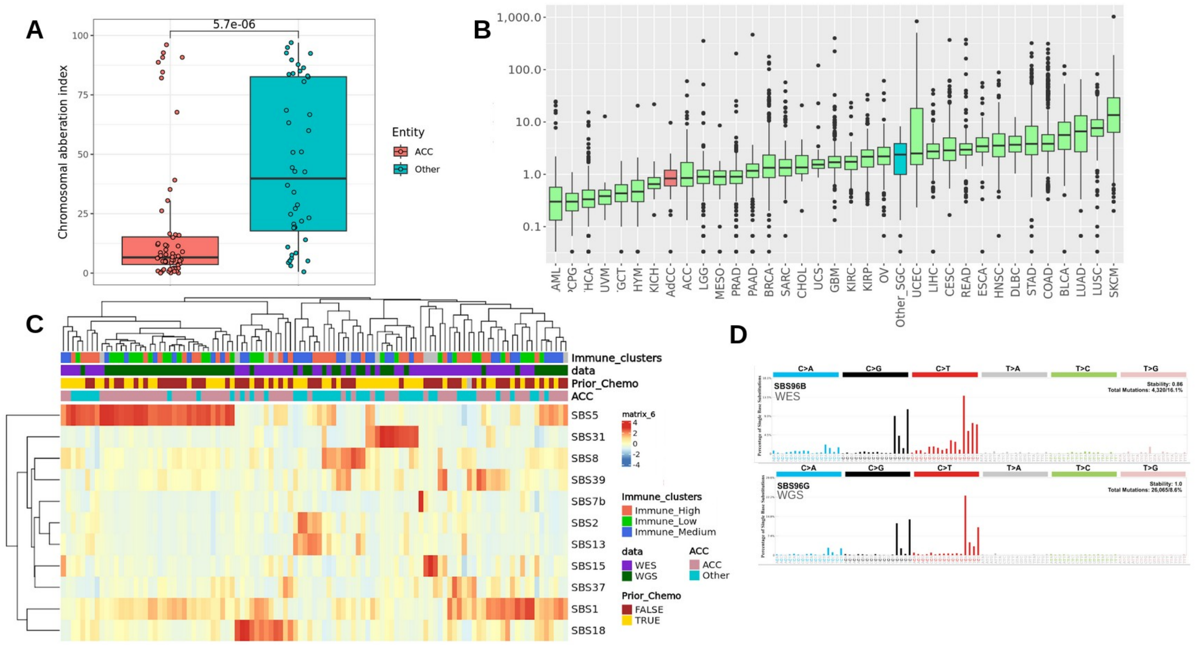

Suppl. Figure 2: *Molecular landscape and mutational signatures.* **A:** Chromosomal aberration index (CAI) of ACC (n=60) versus non-ACC (n=44). **B:** TMB of the MASTER cohort (here ACC was named AcCC to better differentiate from adrenocortical carcinoma) versus all other TCGA cohorts. **C:** Heatmap of SBS signature contributions for recurrent signatures in WES (n=53) and WGS (n=52). Samples were annotated by immune clusters,

tumor entity (ACC/non-ACC), therapy status (prior chemotherapy) and data type (WGS/WES). **D:** De-novo extracted signatures resembling SBS2 and SBS13 in WES and WGS.

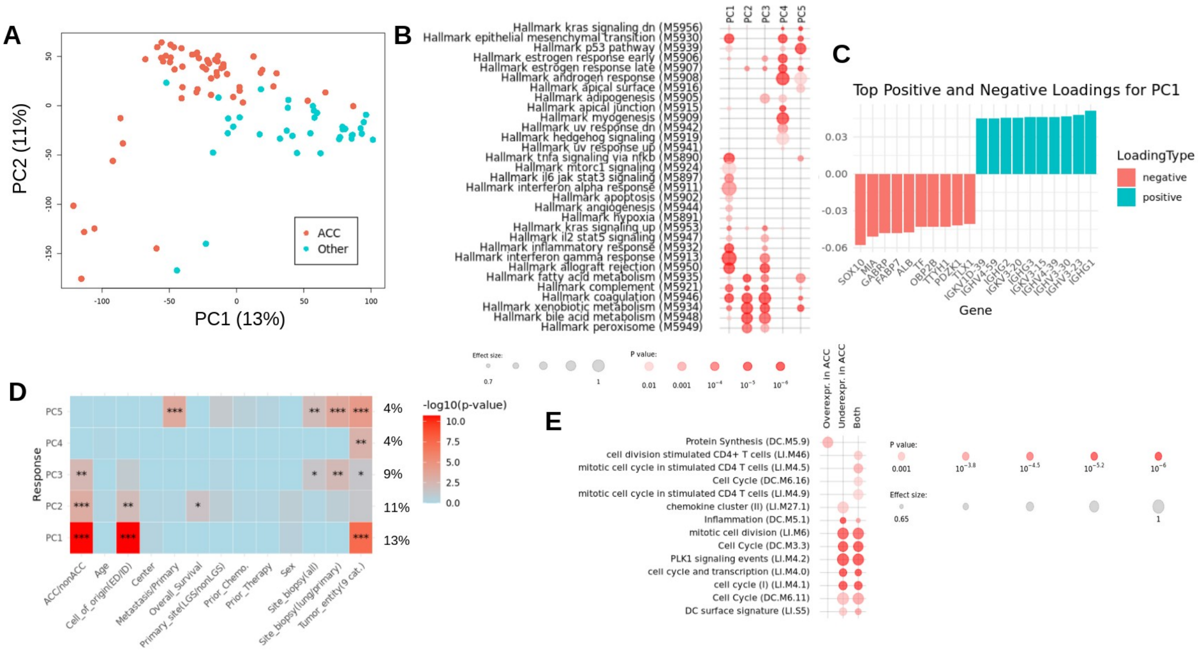

Suppl. Figure 3: *Most variance in transcriptome is explained by tumor entity.* **A:** PCA plot of transcriptome (n=95). Samples were colored by tumor entity (ACC vs Other). **B:** Functional enrichment of first 5 principal components. The size of the dots represents the effect size (AUC) and the color represents the adjusted p-value. **C:** Loading plot for PC1. Plotted are only the top 10 highest and lowest loadings. **D:** Heatmap of adjusted and log-transformed p-values of one-way anovas between the first 5 principal components and clinical variables. **E:** Functional enrichment of differentially expressed genes between ACC and non-ACC. For details see panel B.

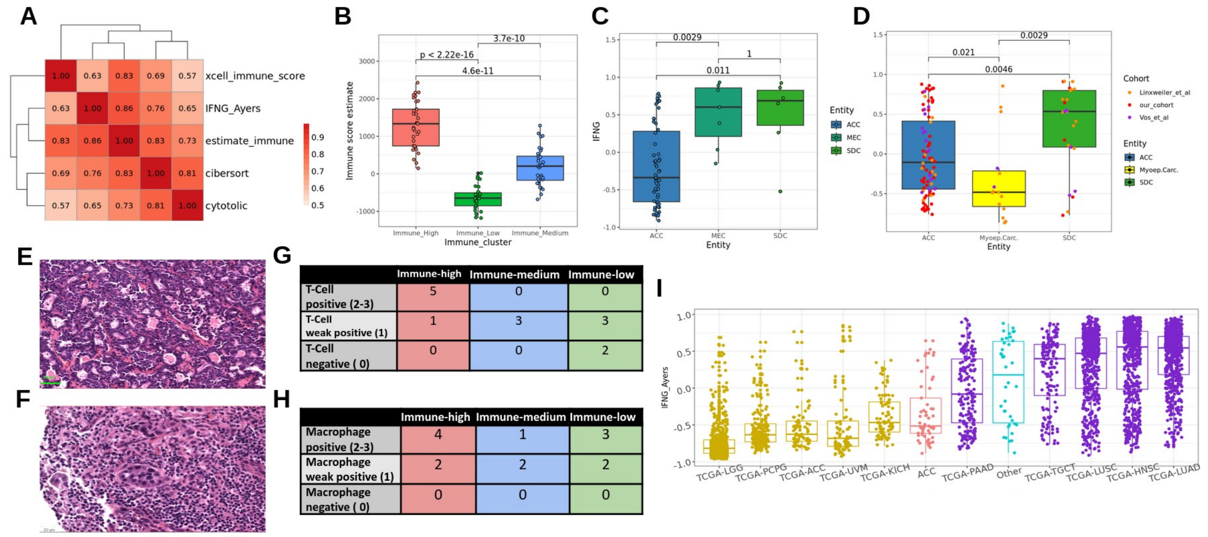

	Immune-high	Immune-medium	Immune-low
T-Cell positive (2-3)	5	0	0
T-Cell weak positive (1)	1	3	3
T-Cell negative (0)	0	0	2

	Immune-high	Immune-medium	Immune-low
Macrophage positive (2-3)	4	1	3
Macrophage weak positive (1)	2	2	2
Macrophage negative (0)	0	0	0

Suppl. Figure 4: *Validation of immune clusters.* **A:** Correlation heatmaps between different measures of immune infiltration based on bulk-data (IFNG GSVA score, cytotolic score, cibersort absolute score, x-cell immune score, estimate immune score) revealed a high overlap between different scores. (*Pearson correlation*, *p-value corrected*) **B:** Estimate immune score grouped by immune clusters (Immune-high n=33; Immune-medium n=34; Immune-low n=28) (*Wilcoxon test*, *two-sided*). **C:** IFNG score grouped by tumor entity with more than 6 samples (ACC n=61; MEC n=7; SDC n=6) in an analysis of the MASTER cohort (*Wilcoxon test, two-sided*). **D:** IFNG score grouped by tumor entity (ACC n= 158; Myoepithelial carcinoma n=26; SDC n=46) with n samples >20 in integrated dataset (*Wilcoxon test*, *two-sided*). **E:** Exemplary staining for CD3 of a sample with low immune-cell infiltration (staining intensity=0). **F:** Exemplary staining for CD3 of a sample with high immune-cell infiltration (staining intensity=3). **G:** Staining for T-cells (CD3+) in 14 samples grouped by intensity and immune clusters as defined by bulk sequencing. **H:** Staining for Macrophages (CD68+) in 14 samples grouped by intensity and immune clusters as defined by bulk sequencing. **I:** IFNG score in MASTER cohort (ACC and Other) versus the top 5 most and least inflamed TCGA cohorts. (PAAD n=183; TGCT n=156; LUSC n=553; HNSC n=566; LUAD n=600; LGG n=534; PCPG n=187; ACC n=79; UVM n=80; KICH n=91).

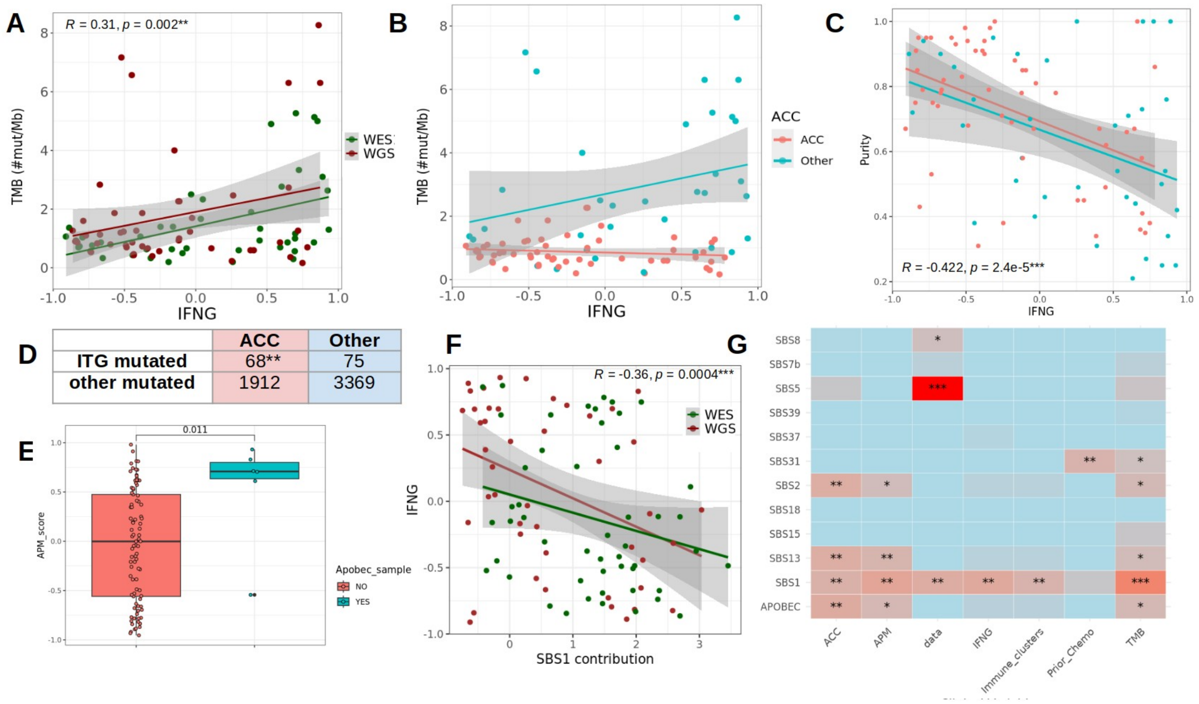

Suppl. Figure 5: *Correlates of immune infiltration.* **A:** TMB vs IFNG score colored by data type (WES n=45; WGS n=50) (*Spearman correlation*)**. B:** TMB vs IFNG score colored by tumor entity (ACC n=58; Other n=37)**. C:** Tumor purity (assessed from WES and WGS data) vs IFNG score colored by tumor entity (ACC n=58; Other n=37) (*Spearman correlation*). **D:** Contingency table of immunotherapy-relevant genes (ITG) mutated in ACC and Other. Mutations in ITG were enriched in ACC samples (*Fisher's exact test*). **E:** APM score in samples with prevalent APOBEC signatures (n=6) and other samples (n=88) (*Wilcoxon test*). **F:** A significantly negative association between SBS1 signature prevalence and the IFNG score was identified. **G:** Heatmap of adjusted and log-transformed p-values of one-way anovas between SBS signature contributions and clinicogenomic parameters (tumor entity: ACC/non-ACC, APM score, data type: WES/WGS, IFNG score, immune clusters, prior chemotherapy and TMB).

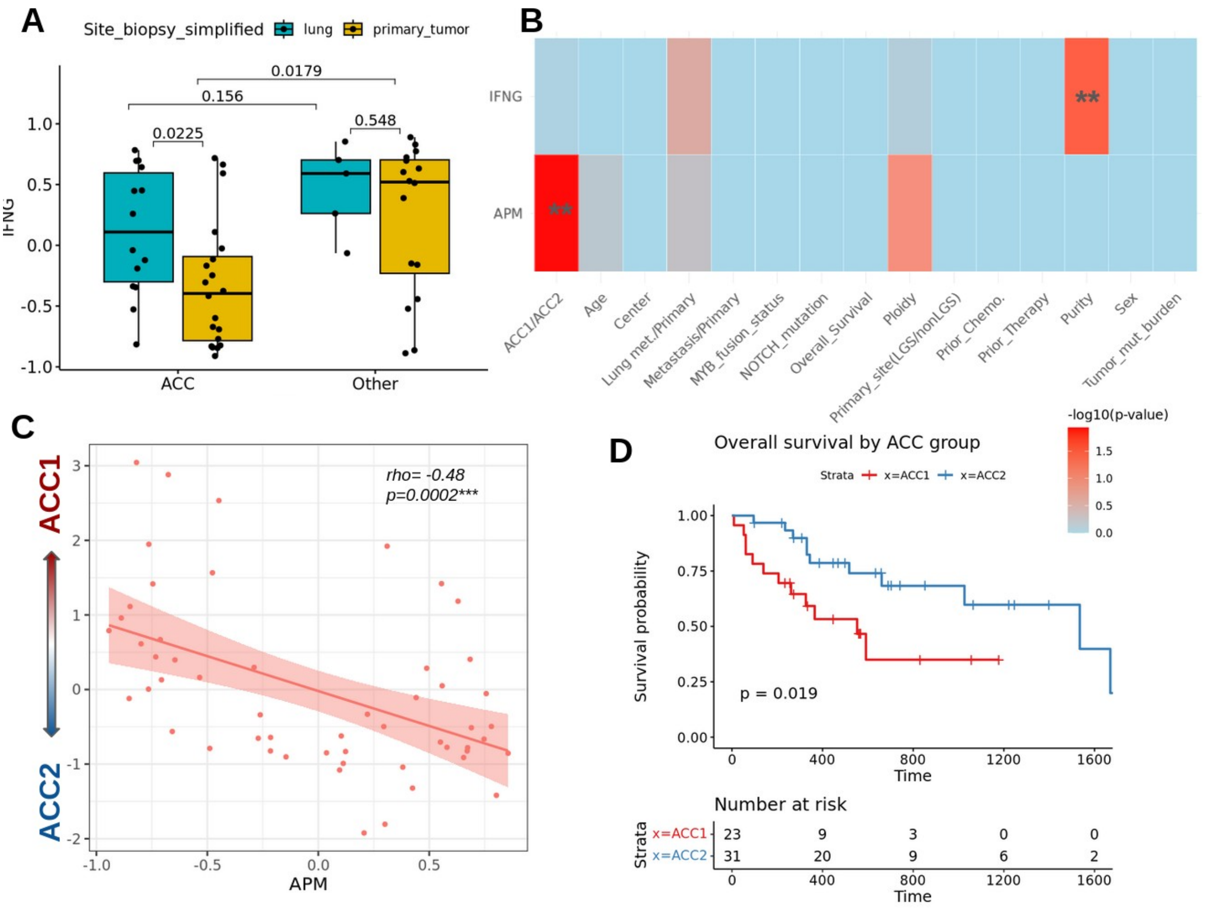

Suppl. Figure 6: *Correlates of immune infiltration in ACC.* **A:** Association of IFNG score with sample origin (lung metastasis vs primary) in ACC (n_lung=13; n_primary=22) and non-ACC (n_lung=8; n_primary=18;) (*Wilcoxon test*). **B:** Heatmap of corrected p-values from One-way anova test of APM/IFNG with clinical variables in ACC samples only. **C:** Correlation plot between ACC-score and APM score (n=57) (*Pearson correlation*)**. D:** Survival plot of ACC1 and ACC2 group *(log-rank test, two-sided).*

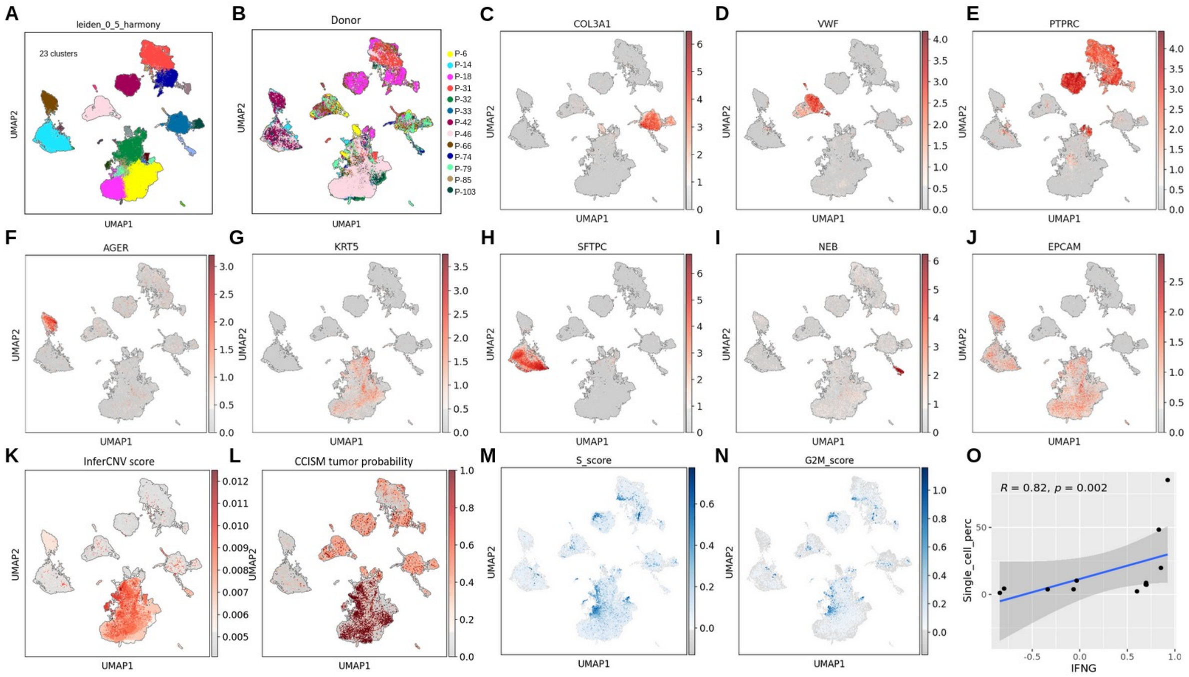

Suppl. Figure 7: *Expression of markers and cell-type annotation in single-nuclei data.* **A and B:** UMAP of integrated single nuclei data colored by clusters (A) and by patient (B). **C-J:** UMAPS with expression of several cell markers (*COL3A1* - Fibroblasts, *VWF* - Endothelial, *PTPRC* - Immune cells, *SFTPC* - Alveolar cells type 2, *AGER* - Alveolar cells type 1, *KRT5* - Basal cells, *EPCAM* - Epithelial cells, *NEB* - Skeletal muscle cells) **K and L:** UMAPS colored by tumor cell probability as calculated by InferCNV (K) and CCISM (L). **M and N:** UMAPS colored by cell cycle scores (S and G2M score). **O:** Correlation plot of immune cell percentage (based on single-cell data) vs IFNG score (based on bulk data) in 13 samples (*Spearman correlation*).

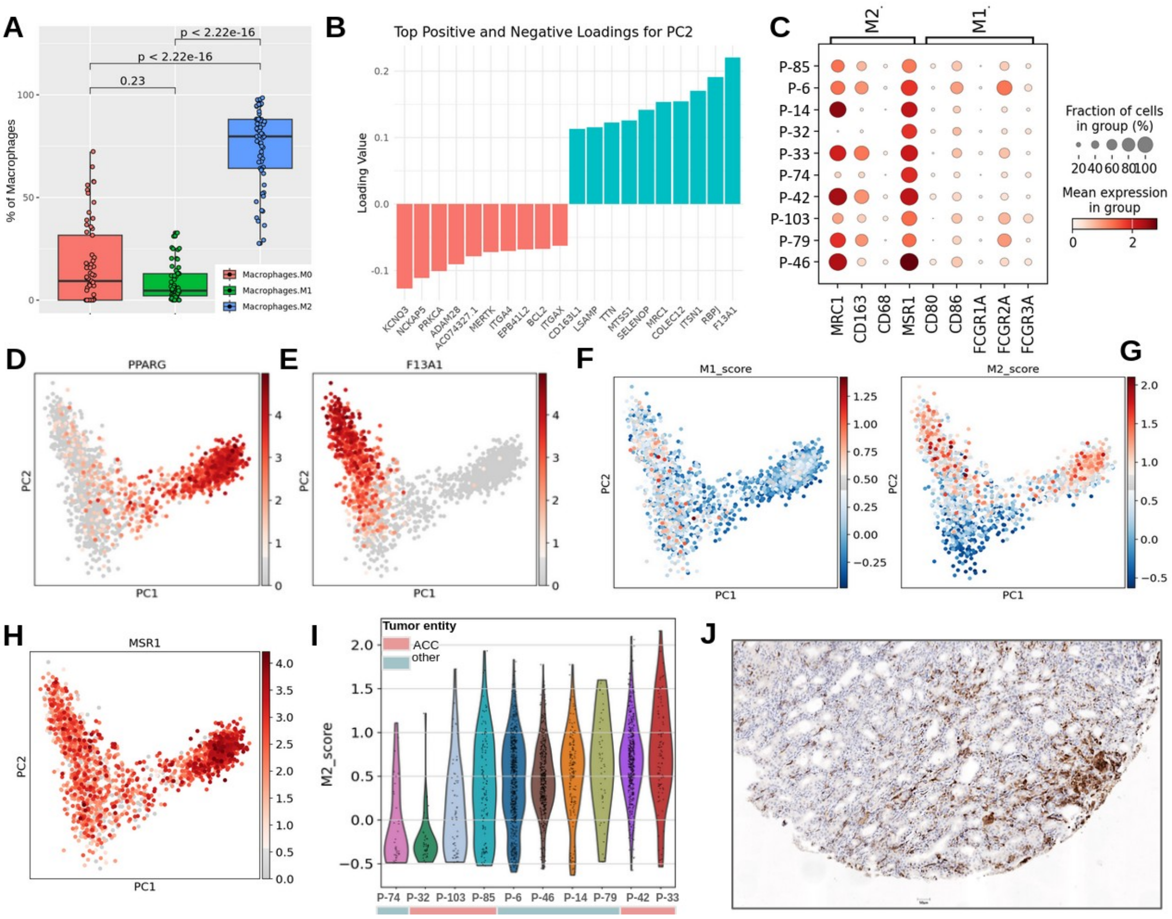

Suppl. Figure 8: *Characterization of tumor infiltrating macrophages.* **A:** Proportions of macrophage subpopulations (M0,M1,M2) relative to total macrophage content in bulk RNAseq (n=61) (*Wilcoxon test*). **B:** PCA loadings for PC2 (single-nuclei sequencing data). **C:** Expression of M2 and M1 markers in macrophages grouped by patient (single-nuclei sequencing data). **D:** PCA plot of macrophages (total cells : 2093, n=10) colored by *PPARG* expression. **E:** PCA plot of macrophages colored by *F13A1* expression in single-nuclei sequencing data. **F and G:** PCA plot of macrophages colored by M1 (F) and M2 (G) score in single-nuclei sequencing data. **H:** PCA plot of macrophages colored by *MSR1* expression in single-nuclei sequencing data. **I:** Violin plot of M2 score in all macrophages grouped by donor in single-nuclei sequencing data. Donors are annotated by tumor entity (ACC/Other). **J:** CD163 (M2 macrophages) staining of an ACC sample.

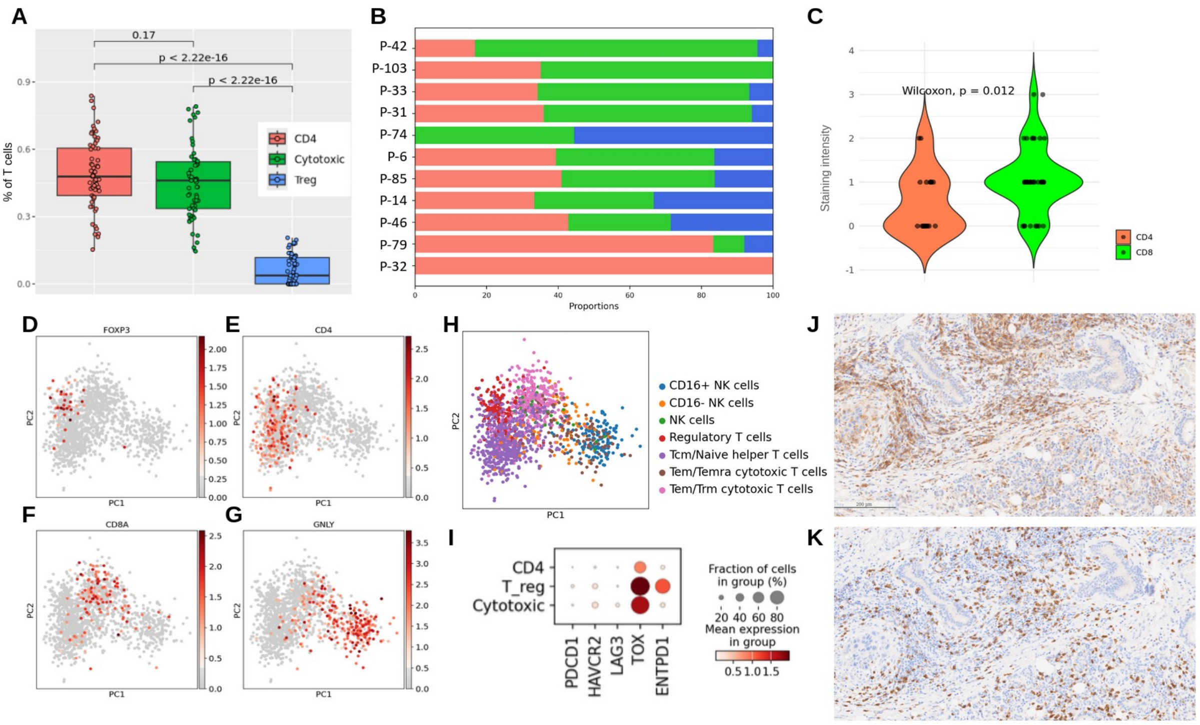

Suppl. Figure 9: *Characterization of tumor infiltrating T/NK-cells.* **A:** Proportions of T/NK cell subpopulations (cytotoxic cells, CD4+ helper T-cells and regulatory T-cells) relative to total T/NK-cell content in bulk RNAseq (n=61) (*Wilcoxon test*). **B:** Proportions of T/NK cell subpopulations (cytotoxic cells, CD4+ helper T-cells and regulatory T-cells) relative to total T/NK cell content in single nuclei data (n=11). **C:** Staining intensity of CD4 (n=18) vs CD8 (n=43). **D-G:** PCA plots of T/NK-cells colored by expression of *FOXP3* (D), *CD4* (E), *CD8A* (F) and *GNLY* (G) (single nuclei data). **H:** PCA plot of T/NK-cells colored by cell type annotation (single nuclei data). **I:** Dotplot showing expression of T-cell exhaustion markers in subpopulations of T/NK-cells (single nuclei data). **J:** Immunohistochemical **s**taining of CD4+ cells in a sample with high T-cell infiltration (Adenocarcinoma NOS). **K:** Immunohistochemical staining of CD8+ cells in the same sample as in panel G (Same scale as figure J).

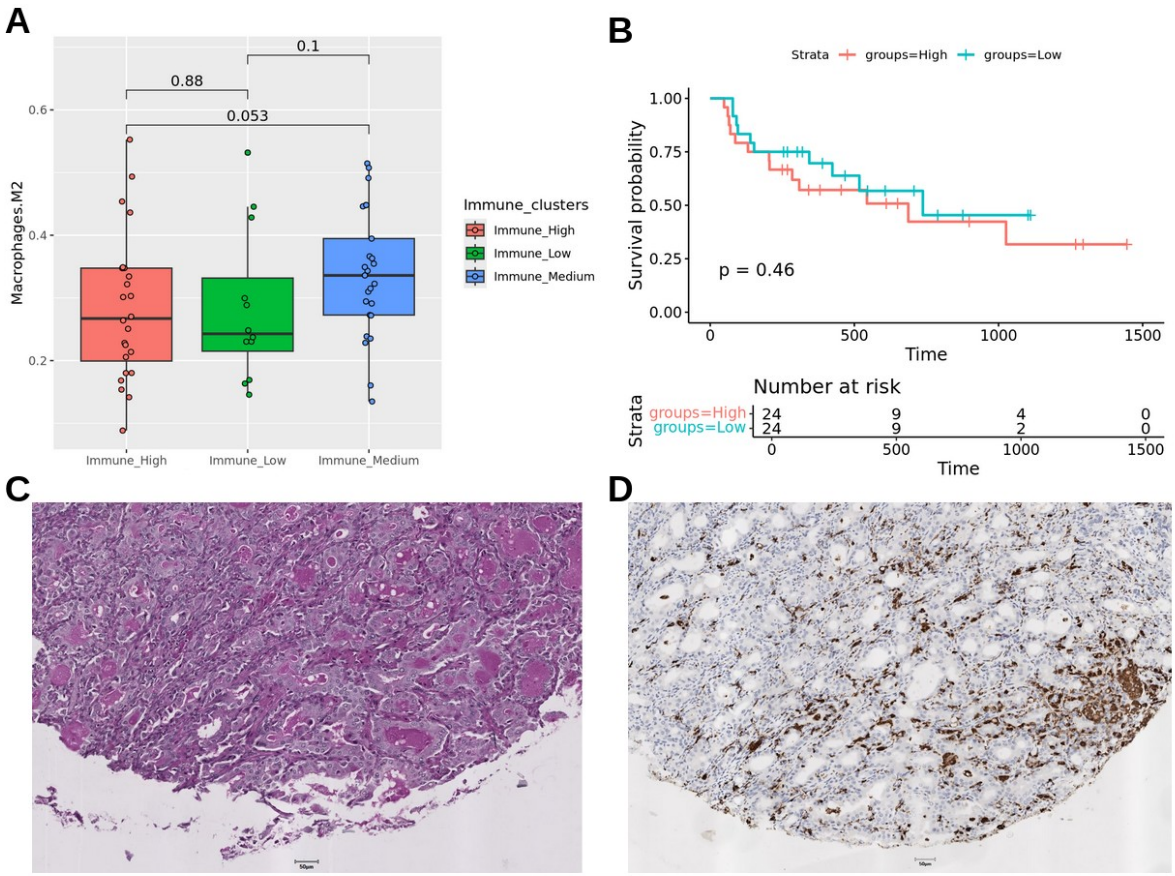

Suppl. Figure 10: *Analysis of macrophages in bulk and IHC.* **A:** Deconvoluted proportions of M2 macrophages in bulk data grouped by immune clusters (p-value filtered, Immune-high n=24; Immune-medium n=25; Immune-low n=12) (*Wilcoxon test*, *two-sided*). . **B:** Survival plot of the samples with the highest and lowest macrophage proportion (upper- and lower quartile of deconvoluted macrophage proportions) *(log-rank test, two-sided).* **C:** Representative depiction of an ACC sample (same sample as in Suppl. Fig 8J) with PAS staining (negative). No muciphages were identified. **D:** CD68 (all macrophages) staining of the same sample as in panel C.

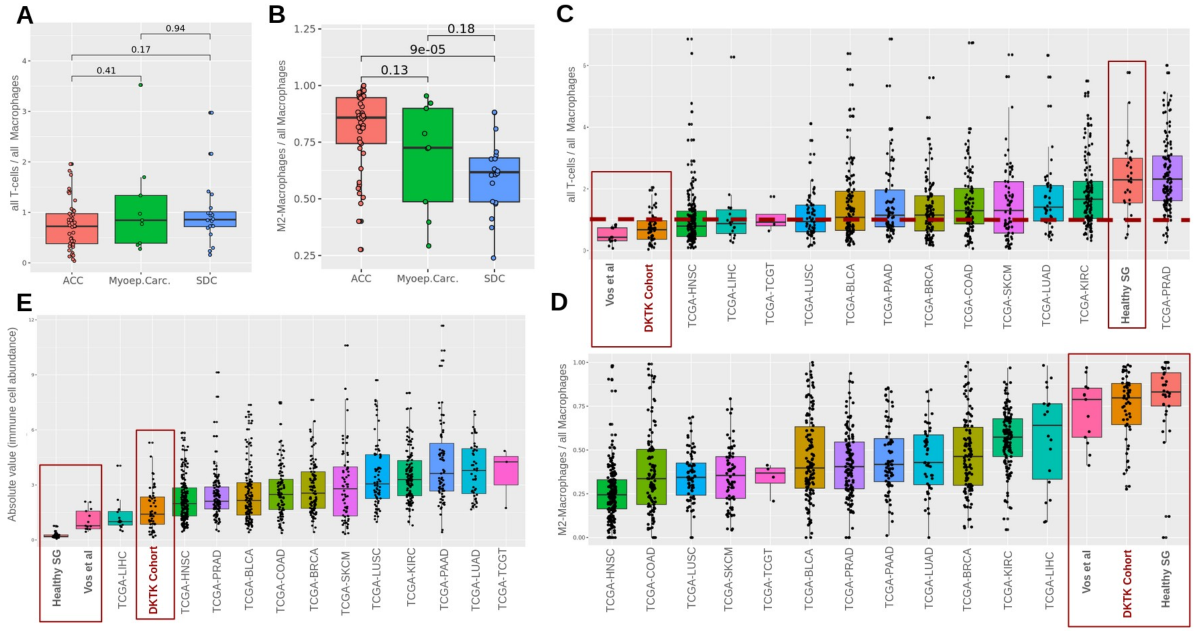

Suppl. Figure 11: *TIM of advanced SGC in the context of TCGA and healthy SG.* **A:** T-cell to macrophage ratio (p-value filtered) in ACC (n=45), myoepithelial carcinoma (n=9), and SDC (n=20) (*Wilcoxon test,two-sided*). Data from integrated cohort. **B:** Relative proportion of M2 macrophages (p-value filtered) in ACC, myoepithelial carcinoma, and SDC (same sample sizes as in A). Data from integrated cohort. **C:** T-cell to macrophage ratio (p-value filtered) in advanced SGC (DKTK MASTER cohort n=61 and Vos et al. cohort n=14 presented separately), 12 TCGA cohorts (BLCA n=141, BRCA n=127, COAD n=126, HNSC n=199, KIRC n=143, LIHC n=21, LUAD n=50, LUSC n=73, PAAD n=88, PRAD n=142, SKCM n=87, TGCT n=4) and healthy SG (n=33). **D:** Relative proportion of M2 macrophages is presented in TCGA cohorts, advanced SGC (DKTK MASTER cohort and Vos et al. cohort presented separately), as well as healthy SG (same sample sizes as in C). **E:** The absolute immune value (immune infiltration) is presented (Healthy SG n=33, Our cohort n=59, TCGA-BLCA n=136, TCGA-BRCA n=122, TCGA-COAD n=116, TCGA-HNSC n=198, TCGA-KIRC n=140, TCGA-LIHC n=20, TCGA-LUAD n=48, TCGA-LUSC n=73, TCGA-PAAD n=82, TCGA-PRAD n=139, TCGA-SKCM n=81, TCGA-TGCT n=4, Vos et al. n=13).

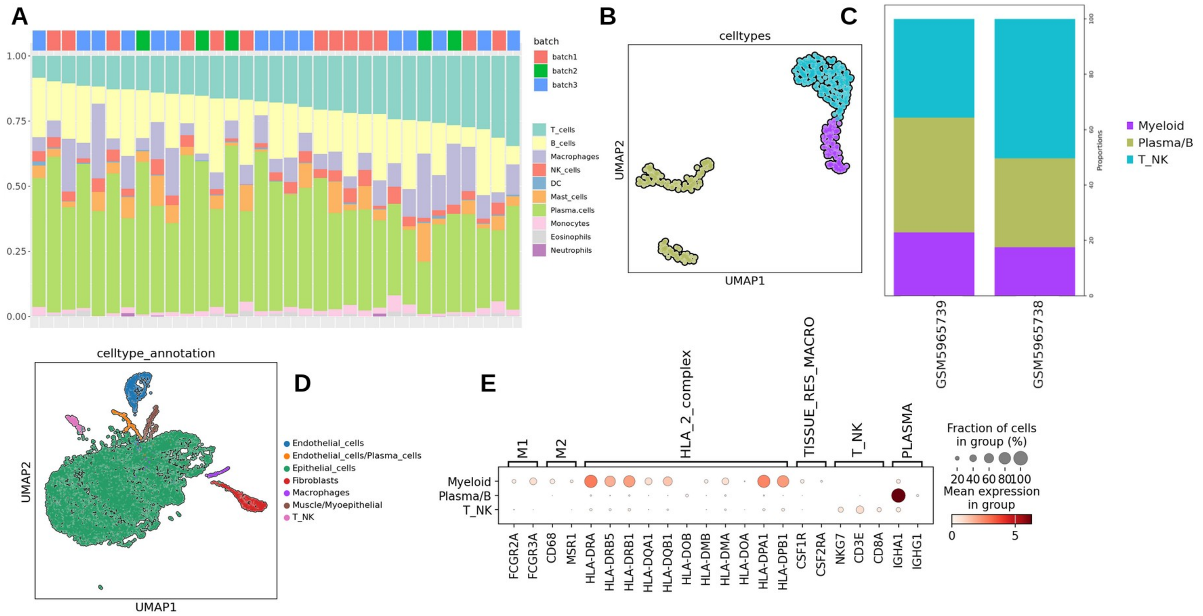

Suppl. Figure 12: *TIM of healthy SG tissue.* **A:** Deconvolution results for 33 samples of healthy SG tissue. Barplot shows proportions of major immune cell subpopulations. Samples were ordered by T-cell proportions and annotated by batch (different cohorts). **B:** UMAP of immune cells in single cell data (n=2). **C:** Proportions of different immune cell subpopulations shown in panel B. **D**: UMAP of integrated data colored by cell types. **E:** Expression of immune cell markers in single cell data.

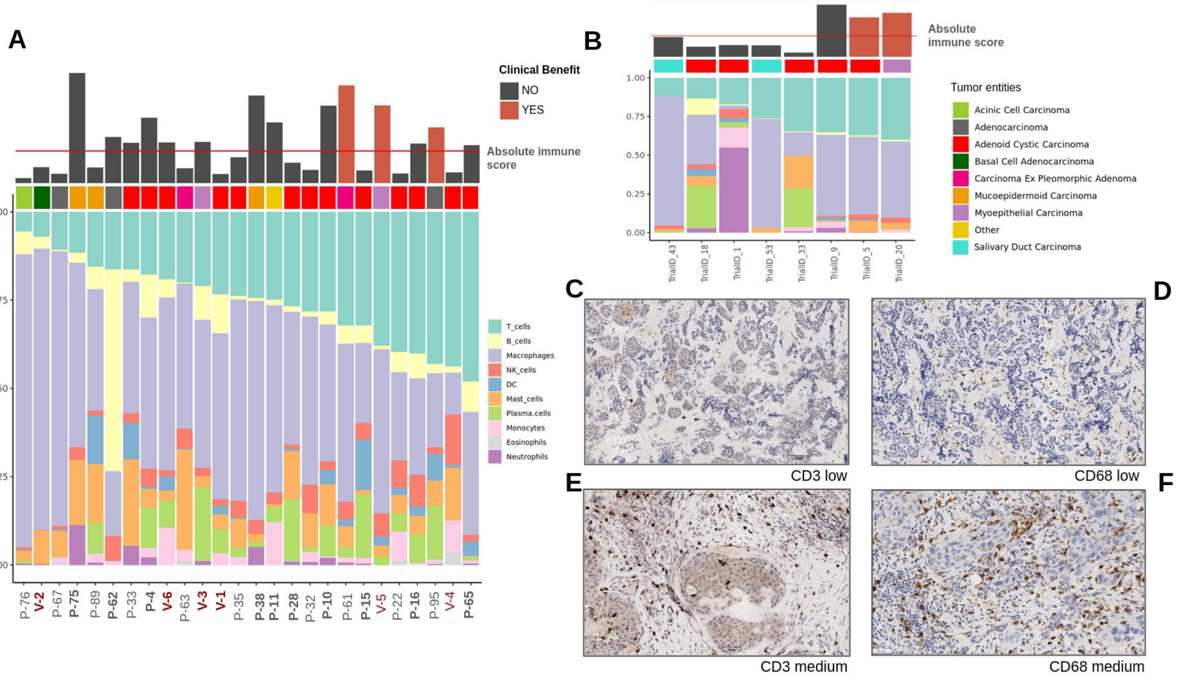

Suppl. Figure 13: *TIM analysis in ICI-samples.* **A:** Deconvolution results of all samples, including those without evaluable results from CIBERSORT deconvolution analysis (n=26)

from the MASTER cohort. The barplots show the proportion of major immune cell subpopulations. The bar on top indicates the absolute immune score. Patients achieving a clinical benefit are highlighted in red. The samples were sorted by T-cell proportion and annotated by tumor entity. **B:** Deconvolution results for 8 samples from the post-treatment Vos et al cohort. For details see panel A. **C:** Example of immunohistochemical staining for CD3 with low intensity. **D:** Example of staining for CD68 with low intensity. **E:** Example of staining for CD3 with medium intensity. **F:** Example of staining for CD68 with medium intensity.

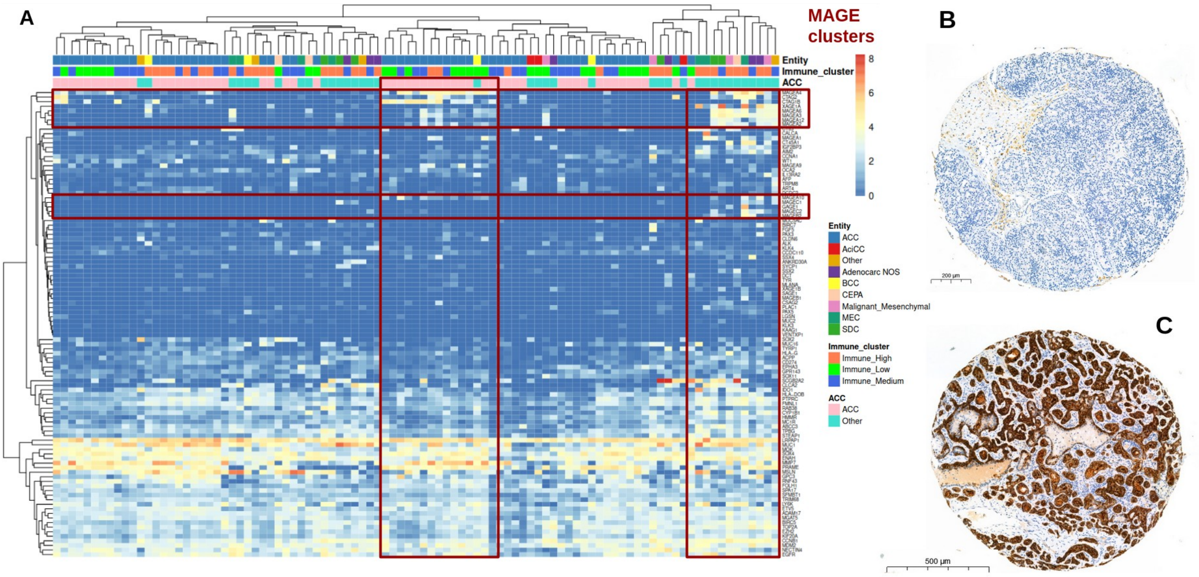

Suppl. Figure 14: *Analysis of biomarkers for immunotherapy.* **A:** Expression of T-cell-target genes (n=95). Samples and genes are clustered. Samples are annotated by Entity and Immune clusters. Marked in red are testis antigens of the MAGE family and samples expressing these genes or some of these genes. **B and C:** Example of *VTCN1* negative (B) and positive (C) staining in two ACC samples.
