# Supplementary material for: A macrophage-predominant immunosuppressive microenvironment and therapeutic vulnerabilities in advanced salivary gland cancer
Source: Nat Commun. 2025 Jun 12;16:5303. doi: 10.1038/s41467-025-60421-0 (PMC12162891; doi:10.1038/s41467-025-60421-0)
Supplement: Supplementary file 2 — Description of Additional Supplementary Files [file 41467_2025_60421_MOESM2_ESM.pdf]

Description of Additional Supplementary Files

Supplementary Data 1:

Clinical and molecular characteristics of the 104 patients included in the study cohort. Clinical variables include sex, age at diagnosis, primary tumor site, tumor entity, presence of metastasis or recurrence, biopsy site, number and type of therapies received prior to biopsy (Ctx: chemotherapy; ICI: immune checkpoint inhibitors; EGFR/HER2-targeted therapy; TKI: tyrosine kinase inhibitors), and the type of next-generation sequencing (NGS) data available. Molecular data include immune cluster assignment, fusion status, and tumor mutational burden (TMB).

Supplementary Data 2:

Results of immunohistochemistry (IHC) analysis in 44 tumor samples. Clinical data include prior exposure to immune checkpoint inhibitors (“Inhibitor”), clinical benefit from ICI, and tumor entity (“Histology”). The IHC markers analyzed include mismatch repair proteins related to Lynch syndrome (MLH1, MSH2, MSH6, PMS2), T-cell markers (CD3 for pan-T cells, CD4 for helper T cells, CD8 for cytotoxic T cells, FOXP3 for regulatory T cells), B-cell and macrophage markers (CD20 for B cells, CD68 for pan-macrophages, CD163 for M2 macrophages, PAS for muciphages), and immune checkpoint proteins (PD-L1 and VTCN1). For PD-L1 and VTCN1, both staining intensity and the percentage of stained cells are reported. Samples labeled with the prefix “IHC” are not part of the main NGS cohort and were included only for validation of IHC findings. Samples marked in orange are part of the ICI validation cohort, while those marked in green are part of the extended IHC analysis.

Supplementary Data 3:

Gene sets used for various transcriptomic and genomic analyses, including the calculation of immune scores in bulk RNA-seq (Figure 3A), enrichment analysis of mutations in immunotherapy-related genes (Supplementary Figure 5D), and cell type-specific gene lists for scoring M1 and M2 macrophages and T cell exhaustion in single-cell RNA-seq data.
