# Supplementary material for: A macrophage-predominant immunosuppressive microenvironment and therapeutic vulnerabilities in advanced salivary gland cancer
Source: Nat Commun. 2025 Jun 12;16:5303. doi: 10.1038/s41467-025-60421-0 (PMC12162891; doi:10.1038/s41467-025-60421-0)
Supplement: Supplementary file 6 — Reporting Summary [file 41467_2025_60421_MOESM6_ESM.pdf]

nature portfolio

Corresponding author(s): Damian T Rieke

Last updated by author(s): Mar 31, 2025

Reporting Summary

Nature Portfolio wishes to improve the reproducibility of the work that we publish. This form provides structure for consistency and transparency in reporting. For further information on Nature Portfolio policies, see our Editorial Policies and the Editorial Policy Checklist.

Statistics

For all statistical analyses, confirm that the following items are present in the figure legend, table legend, main text, or Methods section.

n/a	Confirmed	
☐	☒	The exact sample size (*n*) for each experimental group/condition, given as a discrete number and unit of measurement
☐	☒	A statement on whether measurements were taken from distinct samples or whether the same sample was measured repeatedly
☐	☒	The statistical test(s) used AND whether they are one- or two-sided<br>*Only common tests should be described solely by name; describe more complex techniques in the Methods section.*
☐	☒	A description of all covariates tested
☐	☒	A description of any assumptions or corrections, such as tests of normality and adjustment for multiple comparisons
☐	☒	A full description of the statistical parameters including central tendency (e.g. means) or other basic estimates (e.g. regression coefficient) AND variation (e.g. standard deviation) or associated estimates of uncertainty (e.g. confidence intervals)
☐	☒	For null hypothesis testing, the test statistic (e.g. *F*, *t*, *r*) with confidence intervals, effect sizes, degrees of freedom and *P* value noted<br>*Give P values as exact values whenever suitable.*
☒	☐	For Bayesian analysis, information on the choice of priors and Markov chain Monte Carlo settings
☒	☐	For hierarchical and complex designs, identification of the appropriate level for tests and full reporting of outcomes
☐	☒	Estimates of effect sizes (e.g. Cohen's *d*, Pearson's *r*), indicating how they were calculated

Our web collection on statistics for biologists contains articles on many of the points above.

Software and code

Policy information about availability of computer code

Data collection	Publically available data from the TCGA network were downloaded using the GDC data transfer tool. Additional validation cohorts were downloaded using the SRA toolkit.
Data analysis	No custom code was used.<br>Bulk sequencing data processing: Fastq files were trimmed using bbduk by removing adapter sequences and then mapped using bwa-mem algorithm (version 0.7.17) onto the GRCh38 genome (GRCh38.d1.vd1, primary assembly with decoys and viral sequences). In case of RNA-seq data, alignment and gene expression quantification was performed with salmon (version 1.4.0, transcriptome version: GENCODE 33). Counts were normalized to gene length (TPM) and then used as gene expression measure. To compare gene expression between samples and to run GSVA, counts were “variance stabilized” via vst transformation using the DeSeq2 package in R. Aligned exome and genome data was passed through a somatic variant calling pipeline. SNPs and short indels were detected by the Mutect2 algorithm using in-house panels of normals. The resulting vcf files were annotated via jannovar (version 0.26) and vep (version 102). Gene fusion products were detected with arriba (version 2.3.0). CNVs were computed with Ascat using both exome and genome data.<br>Bulk RNASeq downstream analysis: Differential expression was performed using the R package DeSeq2. DEGs and PCA loadings were tested for functional enrichment against gene sets from MSigDB (release 2023 v1) using the tmod 24 package. The AUC for each enrichment was calculated by ranking genes from the gene set to be tested based on their relevance to a specific module and then assessing how well these ranks separate module-associated genes from non-associated ones. Sample-wise gene set enrichment was performed with GSVA implemented in the R package gsva. Immune cell scores including cytosolic score, ESTIMATE score, x-cell immune score and CIBERSORT score were calculated. CIBERSORT results were validated in independent datasets using the immunedeconv package in R (input: TPM). Batch correction from data integration was done using the limma package in R.<br>Clinical data: Survival analyses were performed with R package survminer.<br>WES/WGS data analysis: Vcf files were converted to maf files by the vcf2maf tool (version 1.6.21) and analyzed by the maftools package in R.

Mutational signatures were computed with MusiCal (SBS mutational signature catalogue v3.4). Tumor purity and ploidy were assessed with Ascat based on WGS and WES.
Single-nuclei sequencing analysis: fastq files were aligned with cellranger (version v 7.1.0). Cellbender was used to filter count matrices and remove background noise. Expected cells and total droplets were estimated from cellranger quality control. Count matrices were processed using the scanpy package in Python. scDBLFInder was used for doublet detection.
Data integration was done using Harmony. For clustering we used the leiden algorithm with 0.5 resolution. Immune cells were annotated automatically using published celltypist. Malignant cells were identified using inferCNV of the Trinity CTAT Project. https://github.com/broadinstitute/inferCNV.

For manuscripts utilizing custom algorithms or software that are central to the research but not yet described in published literature, software must be made available to editors and reviewers. We strongly encourage code deposition in a community repository (e.g. GitHub). See the Nature Portfolio guidelines for submitting code & software for further information.

Data

Policy information about availability of data

All manuscripts must include a data availability statement. This statement should provide the following information, where applicable:

- Accession codes, unique identifiers, or web links for publicly available datasets
- A description of any restrictions on data availability
- For clinical datasets or third party data, please ensure that the statement adheres to our policy

The bulk sequencing data generated in this study have been deposited in the The European Genome-phenome Archive (EGA) with the accession code EGAS50000000809. The single-nuclei sequencing data generated in this study have been deposited in the German Human Genome-Phenome Archive (GHGA) and are accessible through EGA code XXXX. The clinical data generated in this study are provided in the manuscript and supplementary information.

Research involving human participants, their data, or biological material

Policy information about studies with human participants or human data. See also policy information about sex, gender (identity/presentation), and sexual orientation and race, ethnicity and racism.

Reporting on sex and gender	In this study we utilized biological sex rather than gender as a covariate to ensure that our analysis reflects differences that are strictly biological in nature.
Reporting on race, ethnicity, or other socially relevant groupings	In this study we did not include race, ethnicity or other socially relevant groupings as covariates.
Population characteristics	46% of the patients analysed were females and 54% males. The median age was 52 years (IQR 41-61).64% of the patients received a therapy before the sequencing was performed.
Recruitment	The patients in the present study are part of the DKTK Master program, a precision oncology program which aims to characterize and treat incurable cancers.The inclusion criteria are either tumors of rare histology or young age (<51 yrs), and no available standard therapy.
Ethics oversight	Lead Ethics Committee Heidelberg (S-206/2011), Ethics Committee Charité Berlin (EA1/305/21)

Note that full information on the approval of the study protocol must also be provided in the manuscript.

Field-specific reporting

Please select the one below that is the best fit for your research. If you are not sure, read the appropriate sections before making your selection.

☒ Life sciences ☐ Behavioural & social sciences ☐ Ecological, evolutionary & environmental sciences

For a reference copy of the document with all sections, see nature.com/documents/nr-reporting-summary-flat.pdf

Life sciences study design

All studies must disclose on these points even when the disclosure is negative.

Sample size	No statistical method was used to predetermine sample size. The study included all eligible patients with available sequencing data and/or immunohistochemical analyses from the DKFZ/NCT/DKTK MASTER cohort and the retrospective Charité - Universitätsmedizin Berlin cohort. Sample sizes were based on the availability of tumor samples and matched clinical data.
Data exclusions	Samples with insufficient quality, defined by low sequencing quality, incomplete clinical documentation, or technical failure in immunohistochemical staining were excluded. Additionally, for integrated analyses involving multiple cohorts, samples from tumor stages below stage III from published cohorts were excluded to focus specifically on advanced disease.
Replication	Immunohistochemical staining experiments were performed once per marker, per sample, due to limited availability of tumor material. Sequencing experiments were conducted once per patient biopsy, and data reproducibility was assessed by validating results across independent cohorts and multiple analytic modalities (bulk RNA-seq, single-nuclei RNA-seq, immunohistochemistry).

Randomization	Samples were not assigned to experimental groups, available covariates were used to correct for unwanted effects, ensuring robust data analysis and interpretation. The investigators were not blinded to allocation during experiments or outcome assessment.
Blinding	No blinding was required as the nature of the study focused on molecular characterization rather than comparing treatment outcomes or subjective measurements.

Reporting for specific materials, systems and methods

We require information from authors about some types of materials, experimental systems and methods used in many studies. Here, indicate whether each material, system or method listed is relevant to your study. If you are not sure if a list item applies to your research, read the appropriate section before selecting a response.

Materials & experimental systems

n/a	Involved in the study	
☐	☒	Antibodies
☒	☐	Eukaryotic cell lines
☒	☐	Palaeontology and archaeology
☒	☐	Animals and other organisms
☐	☒	Clinical data
☒	☐	Dual use research of concern
☒	☐	Plants

Methods

n/a	Involved in the study	
☒	☐	ChIP-seq
☒	☐	Flow cytometry
☒	☐	MRI-based neuroimaging

Antibodies

Antibodies used	CD3 (polyclonal, solution 1:100, Dako), CD8 (clone C8/144B, solution 1:100, Dako), CD4 (clone 4B12, solution 1:20, Leica) CD20 (clone L26,solution 1:750, Dako) CD68 (clone PG-M1, solution 1:200, Dako), CD163 (clone NCL-L-Cd163, solution 1:400, Leica) FOXP3 (clone 236A/E7, solution 1:200, Abcam plc., Cambridge, UK) PD-L1 (clone E1L3N, solution 1:200, Cell signaling).B7H4/VTCN1 (clone EPR23665-20, Abcam). Antibiodies were validated in an accredited clinical pathology setting.
Validation	Immunohistochemistry was used as validation for available gene expression data from both bulk- and single-cell sequencing. All antibodies were established and tested within a clinical laboratory setting.

Clinical data

Policy information about clinical studies

All manuscripts should comply with the ICMJEguidelines for publication of clinical research and a completedCONSORT checklist must be included with all submissions.

Clinical trial registration	Registrational, non-interventional. Registration: NCT05852522
Study protocol	No interventional trial. No outcome measures.
Data collection	The DKTK MASTER program applies comprehensive molecular diagnostics to inform the care of adult patients with incurable cancers. DKTK MASTER inclusion criteria were advanced solid tumors of a rare histology or younger age (< 51y), no available standard therapy, and available fresh-frozen tumor tissue. No other selection bias. Multicentric recruitment of patients in a national precision oncology program.
Outcomes	no primary outcome measures.

Plants

Seed stocks	N/A
Novel plant genotypes	N/A
Authentication	N/A
